# Dr. Frank Merril Vebber

**Published:** 1914-12

**Authors:** 


**Dr. Frank Merril Vebber,** Eclectic of New York, 1884, of Clayton, N. Y., died in the Watertown City Hospital, of diabetes, October 20, aged 58.

